# Estimating the Fitness Cost of Escape from HLA Presentation in HIV-1 Protease and Reverse Transcriptase

**DOI:** 10.1371/journal.pcbi.1002525

**Published:** 2012-05-24

**Authors:** Rafal Mostowy, Roger D. Kouyos, Ilka Hoof, Trevor Hinkley, Mojgan Haddad, Jeannette M. Whitcomb, Christos J. Petropoulos, Can Keşmir, Sebastian Bonhoeffer

**Affiliations:** 1 Institute for Integrative Biology, ETH Zurich, Zurich, Switzerland; 2 Department of Ecology and Evolutionary Biology, Princeton University, Princeton, New Jersey, United States of America; 3 Theoretical Biology/Bioinformatics, Utrecht University, Utrecht, The Netherlands; 4 Monogram Biosciences, South San Francisco, California, Unites States of America; La Jolla Institute for Allergy and Immunology, United States of America

## Abstract

Human immunodeficiency virus (HIV-1) is, like most pathogens, under selective pressure to escape the immune system of its host. In particular, HIV-1 can avoid recognition by cytotoxic T lymphocytes (CTLs) by altering the binding affinity of viral peptides to human leukocyte antigen (HLA) molecules, the role of which is to present those peptides to the immune system. It is generally assumed that HLA escape mutations carry a replicative fitness cost, but these costs have not been quantified. In this study, we assess the replicative cost of mutations which are likely to escape presentation by HLA molecules in the region of HIV-1 protease and reverse transcriptase. Specifically, we combine computational approaches for prediction of *in vitro* replicative fitness and peptide binding affinity to HLA molecules. We find that mutations which impair binding to HLA-A molecules tend to have lower *in vitro* replicative fitness than mutations which do not impair binding to HLA-A molecules, suggesting that HLA-A escape mutations carry higher fitness costs than non-escape mutations. We argue that the association between fitness and HLA-A binding impairment is probably due to an intrinsic cost of escape from HLA-A molecules, and these costs are particularly strong for HLA-A alleles associated with efficient virus control. Counter-intuitively, we do not observe a significant effect in the case of HLA-B, but, as discussed, this does not argue against the relevance of HLA-B in virus control. Overall, this article points to the intriguing possibility that HLA-A molecules preferentially target more conserved regions of HIV-1, emphasizing the importance of HLA-A genes in the evolution of HIV-1 and RNA viruses in general.

## Author Summary

Our immune system can recognize and kill virus-infected cells by distinguishing between self and virus-derived protein fragments, called peptides, displayed on the surface of each cell. One requirement for a successful recognition is that those peptides bind to the human leukocyte antigen (HLA) class I molecules, which present them to the immune system. As a counter-strategy, human immunodeficiency virus type 1 (HIV-1) can acquire mutations that prevent this binding, thereby helping the virus to escape the surveillance of T-lymphocytes. It is likely that the virus pays a replicative cost for such escape mutations, but the magnitude of this cost has remained elusive. Here, we quantified this fitness cost in HIV-1 protease and reverse transcriptase by combining two computational systems biology approaches: one for prediction of *in vitro* replicative fitness, and one for the prediction of the efficiency of peptide binding to HLA. We found that in viral proteins targeted by HLA-A molecules, mutations which disrupt binding to those molecules carry a lower replicative fitness than mutations which do not have such an effect. We argue that these results are consistent with the hypothesis that our immune systems might have evolved to target genetic regions of RNA viruses which are costly for the pathogen to alter.

## Introduction

The evolutionary dynamics of viral infections are often characterized by the opposing forces of immune control and viral escape. These forces shape both the within-host dynamics of infections as well as the dynamics of spread on an epidemiological level. At the within-host level, the role of the opposing forces are manifest in chronic infections such as HIV, SIV, and HCV, where it has been shown that the virus population frequently escapes immune control by B- or T-cell responses [1]–[8]. Moreover, in HIV/SIV, slow disease progression is associated with efficient immune control via protective human leukocyte antigen (HLA) genes; see [9]–[11], and escape events can precipitate the loss of immune control [3], [12], [13]. At the epidemiological level, the selection for escape imposed by the host population can be traced in the genetic structure of viral infections [14]–[17].

The balance between immune control and escape often affects the success of vaccines. Vaccines against viral infections characterized by complete absence of escape, such as smallpox, measles, mumps and rubella, are typically highly protective [18], whereas vaccines against viruses which periodically escape immune control, such as influenza, require periodic updating [19]–[21]. In HIV-1, antigenic diversity, often stemming from escape at the within-host level, represents a formidable obstacle for any potential vaccine to overcome [22]–[24].

Deeper insight into the coevolutionary dynamics of immune control and viral escape requires an improved quantitative understanding of the benefits and costs of escape from the immune responses. The literature on HIV-1 and SIV escape clearly documents the existence of costs and benefits for viral escape as well as the necessity of quantifying them. A good example is the escape from the presentation by major histocompatibility complex (MHC) class I molecules, in humans encoded by HLA genes [3]. Furthermore, escape mutations often revert in HLA-mismatched patients [25]–[27] and they are often followed by compensatory mutations [28]–[30], suggesting that escape mutations are costly. Large variability in the cost of escape mutations has been demonstrated experimentally [31], and mathematical models have shown that the dynamics of immune escape strongly depend on the cost of escape mutations both at the within-host and the epidemiological level [32]–[34]. Taken together these studies underline the importance of assessing the replicative cost of escape mutations.

In this study, we quantified the fitness cost of those mutations in HIV-1 protease (PR) and reverse transcriptase (RT) which are likely to escape the presentation of HLA molecules using an *in silico* approach, focusing on escape from presentation by HLA-A and HLA-B molecules. To this end, we analyzed a dataset of more than 70'000 genetic sequences of HIV-1 PR and RT from the North American population. Specifically, we combined two computational approaches: First, we used the results of a ridge-regression approach to quantify the impact of single amino acid substitutions in the region of interest on viral replicative capacity *in vitro* [35]. Second, the impact of these mutations on binding affinity to various HLA molecules was predicted using a neural network approach, as implemented in [36], [37]. Combining these two methods we examined whether mutations which are more likely to disrupt binding to HLA molecules carry a higher replicative cost *in vitro* than other mutations.

## Materials and Methods

### Data

Over 70'000 virus sequences from HIV-1 subtype B were assayed for the replicative capacity of the Pol gene, specifically the entire protease (PR) and positions 1 to 305 of the reverse transcriptase (RT). The assay is described in detail in ref. [38]. In brief, the region of interest (404 amino acids) was inserted into a viral backbone derived from the NL4-3 molecular clone. The NL4-3 clone was modified so that it can go through a single round of replication. Replicative capacity for each genetic variant was then assessed *in vitro* by measuring the total infectious progeny virus produced after a single round of replication.

### Quantification of fitness costs

The quantification of fitness costs was based on a method described in detail elsewhere [35]. In short, the data described above were fitted to a fitness model of single and double mutations to estimate the effect of these mutations on the viral replicative capacity *in vitro* using a machine-learning approach (generalized kernel ridge regression). This fitness prediction model had previously been trained on a data set of 65,000 sequences and had been cross-validated on 5,000 sequences, where it explained between 35% and 66% of the variance in fitness depending on the environment in which replication was measured. Specifically, the model used here is based on the estimated main effects and pairwise epistatic effects of the 1'848 amino acid variants found in these sequences, and therefore can predict the fitness of all 1'848 single non-synonymous mutations which naturally occurred in the viral population derived from North American patients.

Here we used this predictive model to calculate the fitness effects of single mutants, which were generated by substituting a single amino acid in the wild-type NL4-3 sequence. The relative fitness of such a single mutant was then given by its predicted replicative capacity relative to the predicted replicative capacity of the NL4-3 sequence. The predicted fitness of the NL4-3 sequence was therefore equal to 1.

### Epitope prediction

In order to achieve broad HLA coverage, we used the pan-specific method NetMHCpan2.4 [36], [37] to predict binding affinities of peptides to HLA class I molecules. This machine-learning method (neural network approach) had been trained on a large set of quantitative MHC-peptide binding data for more than 80 human and non-human MHC class I molecules (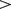
90% human). The method extrapolated the “rules” of MHC-peptide binding from the training set to predict the binding affinity of MHC:peptide combinations that were not part of the training process. In this study we focused on two of three HLA class I loci: HLA-A and HLA-B. In a benchmark study based on these two genes, NetMHCpan was shown to outperform other pan-specific prediction methods [39]. Binding predictions to HLA-C were ignored because of performance issues.

To utilize NetMHCpan to predict epitopes in PR (amino acids 1–99) and RT (amino acids 1–305), we cleaved the corresponding amino acid sequence of NL4-3 into all possible peptides of length 9 (9mers). We quantified the binding affinity for each peptide:HLA combination by predicting a binding affinity of the complex, which was characterized by an 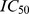 value (concentration at which half-maximal inhibition in the assay was achieved). A low 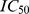 score translated into a high binding affinity, and a high 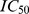 score translated into a low binding affinity. A peptide was then considered an epitope if its predicted binding affinity for a given HLA molecule exceeded either a relative or an absolute threshold. According to the absolute criterion, a peptide was an epitope for a given HLA allele if the obtained 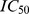 score was lower than 500 nM, as suggested experimentally [40]. According to the relative criterion, a peptide was an epitope for a given HLA allele if the obtained 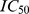 score fell within the bottom 1% of all scores obtained for this HLA allele based on a large set (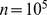) of random natural peptides. Both binding definitions are widely used in the literature [41]–[43].

### Impairment of binding affinity in mutated epitopes

The degree to which a single mutation in a peptide impairs its binding to an HLA allele is considered here only if the peptide was an epitope for the given allele based either on the relative or the absolute criterion. Hence, assessing binding impairment depends on the criterion used to predict epitopes. The impairment of binding affinity due to single mutation in epitopes was defined as
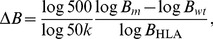
(1)where 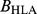 is the 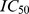 binding threshold to a HLA molecule in question, 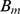 is the 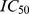 score of the mutated peptide to this HLA molecule, 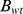 is the 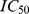 score of the wild-type (non-mutated) peptide to this HLA molecule, and 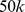 denotes the value of 50,000 nM considered a maximal experimental sensitivity threshold. If multiple HLA molecules were affected by a single mutation, the strongest impairment was taken into account. Note that for the absolute criterion used here, the multiplication factor in the front reduces to 1, as 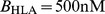 for each HLA molecule.

### Frequency of HLA-alleles and escape

The frequency of a given HLA allele in the US population was estimated from a HLA haplotype study in the US population stratified by ethnicity [44]. The HLA alleles used for this study consisted of all alleles for which we obtained frequency data and which featured in netMHCpan2.4, giving overall 98 HLA-A alleles and 184 HLA-B alleles. An expected frequency of each given HLA allele was calculated as the mean ethnicity-stratified frequency, weighted by the HIV-1 prevalence estimates in the US population for different ethnicities [45]. The list of all HLA alleles used in this study, together with the calculated frequency values are given in Table S1.

To investigate the role of the selective pressure at the population level, we defined a ‘mutation impact’ and a ‘population-weighted mutation impact’, given by
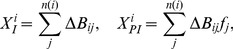
(2)where 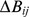 is an impairment of binding HLA molecule 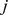 due to mutation 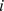, 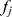 is the expected frequency of HLA 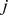 in the population, and 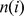 is the number of HLA molecules binding to the wild-type; all values characterized by 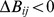 were ignored.

We classified HLA alleles that occur at frequencies of 0.5% or lower in the host population as infrequent, assuming that these HLA alleles impose negligible selection pressure for escape in the host population. In the analysis of population-independent effects we removed all frequent HLA alleles from the analysis.

### Protective and non-protective alleles

To identify protective and non-protective HLA alleles, we assigned a relative hazard for progression to AIDS (RH) to each HLA molecule, based on a survival analysis of HIV-1 positive patients from an earlier study [46]. HLA alleles with a low RH value tend to be associated with long-term non-progression to AIDS, while HLA alleles with a high RH value tend to be associated with rapid progression to AIDS. As the relative hazard values in this study had been associated with low resolution HLA molecules (two-digit, e.g., HLA-A*02) rather than with high-resolution HLA molecules (four-digit, e.g., HLA-A*02:01), in the analysis we assigned the relative hazard values to all HLA alleles of a particular group. For example, the 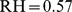, associated with the allele HLA-A*26, was assigned to HLA-A*26:01, -A*26:02, -A*26:03, -A*26:07, -A*26:08, -A*26:09, and -A*26:12. In our analysis, protective HLA alleles were defined as the 10 alleles (according to the two-digit classification) with the lowest RH value (HLA-B*58, -B*27, -B*57, -A*26, -B*51, -A*11, -A*32, -B*13, -B*14, -A*31), and non-protective HLA alleles were defined as the 10 alleles (according to the two-digit classification) with the highest RH value (HLA-A*66, -A*74, -B*35, -B*53, -B*45, -B*50, -A*33, -B*39, -A*68, -B*56). According to the four-digit classification this corresponds to 54 protective HLA alleles and 60 non-protective alleles.

### Statistical analysis

The non-parametric Mann-Whitney or Wilcoxon signed-rank test was used for statistical comparisons of data samples with measured replicative fitness. The non-parametric Spearman rank test was used to calculate correlation coefficients between fitness and impairment of binding, with the confidence intervals calculated using the Fisher transformation. Results with 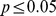 or less were considered significant; results with 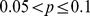 were considered as showing a trend of significance. All correlations and data analyses were performed using the R software package [47].

## Results

**Figure 1:**
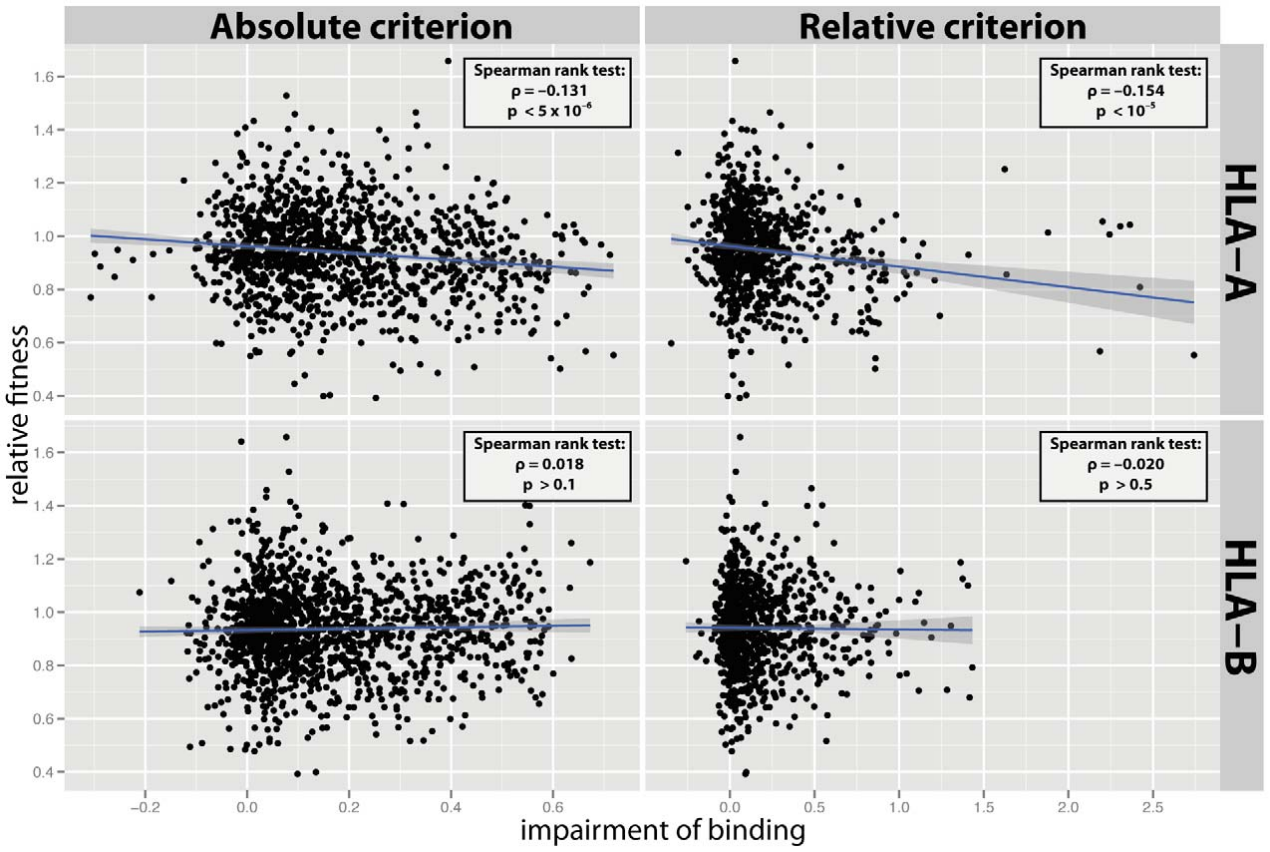
Cost of mutations which impair the HLA-binding. (**Top row**) Effect of mutations on binding affinity to HLA-A molecules according to two alternative epitope definitions (absolute criterion: 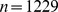, relative criterion: 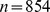). In both cases we observed a significant correlation between the fitness of single mutants and the impairment of binding to HLA-A (absolute criterion: 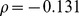, 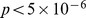; relative criterion: 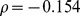, 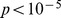). Each datapoint corresponds to a single amino acid substitution in the genetic region restricted by HLA alleles of the corresponding locus, A or B. Note, that if multiple HLA molecules were affected by a single mutation at a given locus, the strongest impairment was plotted here. (**Bottom row**) Effect of mutations on binding affinity to HLA-B molecules for the two alternative epitope definitions (absolute criterion: 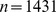, relative criterion: 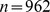). Here, no significant correlation between the quantities in question was found (absolute criterion: 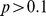; relative criterion: 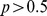). For the sake of illustration, the blue line shows the best fit of a linear regression and the 95% confidence interval.

In order to test whether the fitness cost of single mutations is associated with their impact on HLA presentation, we analyzed the correlation between the predicted relative fitness of single mutations and their effect on HLA binding affinity. The *in vitro* fitness of mutations (replicative capacity) was predicted via ridge regression [35] and their impact on HLA presentation was predicted via a neural-networks approach [37]; see Materials & Methods. We first assessed the relation between the fitness of mutations and their effect on HLA binding. To that end, we quantified the impairment of binding caused by each mutation as a log of the ratio of binding affinity prior to the mutation and after the mutation, as given by equation (1). The analysis was performed once for HLA-A molecules, and once for HLA-B molecules. Specifically, in the analysis for HLA-A we considered all mutations in epitopes restricted by HLA-A molecules, and in the analysis for HLA-B we considered all mutations in epitopes restricted by HLA-B molecules (even though these sets of mutations may overlap). If, for a given HLA locus (A or B), a mutation was part of more than one epitope, the strongest effect was considered for that HLA locus, ensuring that one mutation gave rise to a single data point per HLA locus. Fig. 1 shows a correlation between the predicted fitness of single mutations and their impact on binding affinity to HLA-A and HLA-B molecules. The disruptive effect of mutations on binding between HIV-1 peptides and HLA-A molecules was found to correlate significantly with the fitness of those mutations, independent of the epitope definition employed (absolute criterion assumes same binding threshold for each HLA molecule; relative criterion assumes that each HLA molecule binds a similar number of peptides; see Materials & Methods), however no such correlation was observed for HLA-B molecules (HLA-A: absolute criterion: 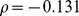, 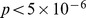; relative criterion: 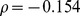, 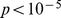; HLA-B: absolute criterion: 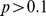; relative criterion: 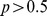). Thus, mutations which disrupt binding to HLA-A molecules seem to carry a higher fitness cost than mutations that do not.

To assess whether this association holds independently of the binding criterion employed, we examined the association between fitness and change in binding for a published list of experimentally defined optimal CTL epitopes and the corresponding HLA molecules [48], which defined the used peptide:HLA complexes. We observed a trend of a negative correlation between fitness and binding impairment when mutations in peptides restricted by both HLA-A and HLA-B were considered (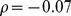, 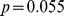, 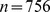), however no signal was detected for individual correlations in HLA-A (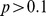, 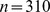) or HLA-B (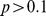, 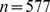). Although the first result seems to support the notion of a negative correlation between replicative fitness and binding impairment to HLA molecules in the HIV-1 region examined, it remains not fully clear whether the lack of a strong signal is due to a low number of HLA:peptide pairs provided in the list of best-defined CTL epitopes (81 instead of 1850 and 796 predicted ones for the absolute and relative criterion, respectively), or due to any problems associated with the classification of optimal epitopes.

**Figure 2:**
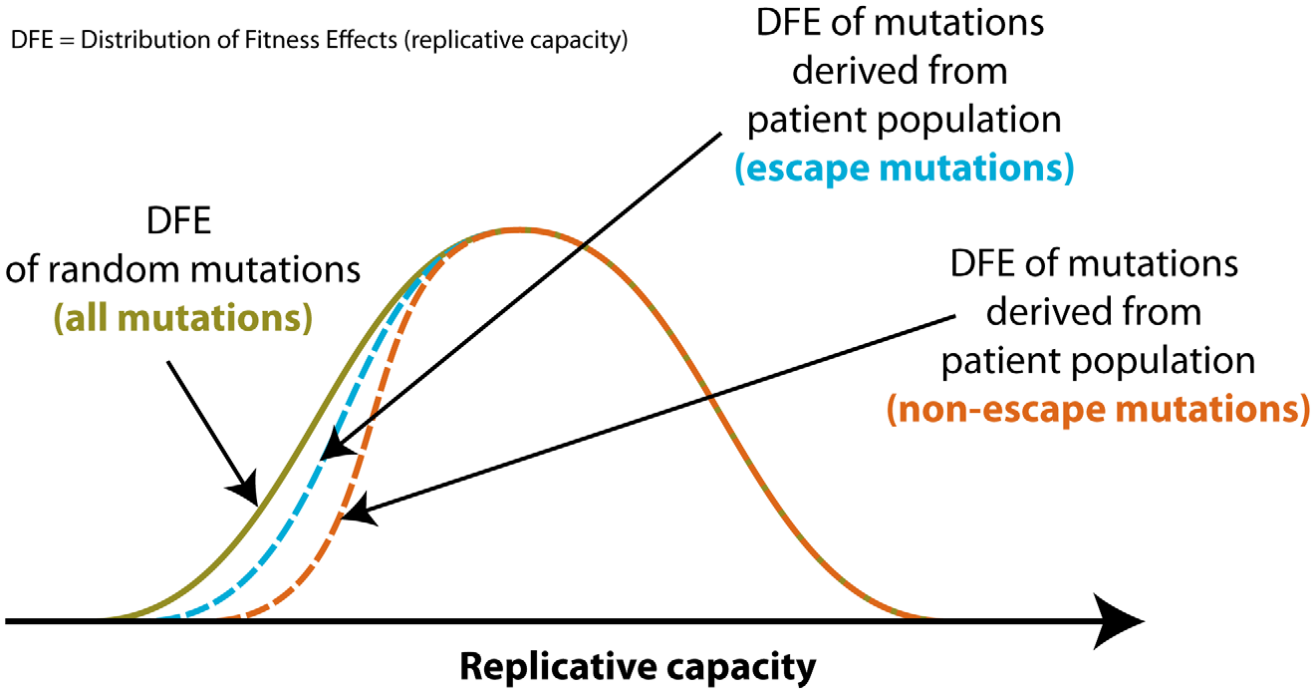
Population effect in the observed cost of escape from HLA presentation. The distribution of replicative fitness effects (DFE) of random mutations in HIV-1 (green) will differ from the corresponding distribution of mutations derived from the patient population (blue/orange) as the latter will feature only mutations which have undergone selection. Mutations with a particularly high replicative cost (i.e., low replicative capacity) will have a low probability of being present in the patient-derived data because they will persist at very low frequencies in the virus population. However, escape mutations carry an additional benefit of avoiding being killed by the immune system and therefore are expected to have a higher *in vivo* fitness, which allows for persistence in spite of a lower replicative capacity. For this reason, even if the DFE of escape and non-escape mutations are identical, the escape mutations derived from the patient population (blue) may appear to have on average a lower replicative capacity than the non-escape mutations derived from the patient population (orange).

The association between the effect of a mutation on fitness and its predicted effect on HLA-A binding could be due to two, mutually not exclusive, factors. First, there might be a population effect caused by selection for escape at the host population level, as shown in Fig. 2. Since our data are not based on random mutations but mutations as they are observed in viral populations sampled from the infected host population, we would expect mutations with a high replicative cost (i.e., low replicative capacity *in vitro*) to be underrepresented in our dataset. However, for escape mutations such replicative cost could be offset by the benefit of conferring escape, especially if the mutant confers escape in a large fraction of the host population. For this reason, two mutations of the same replicative capacity, one escaping a large fraction of the host population and another escaping none, would not spread evenly in the host population because the escape mutation would have a higher *in vivo* fitness than the non-escape mutation, and consequently the escape mutation would be present at a higher frequency in the population than the non-escape mutation. The resulting excess of escape mutations with high replicative cost would manifest itself in the data as a difference in relative fitness *in vitro* between the two types of mutations (see also Fig. 2). We expect this effect to be the most dramatic for mutations with high replicative cost because mutations with a low replicative cost will most likely occur in the dataset even if they carry no additional advantage against the immune response. Second, there could be an intrinsic effect stemming from intrinsic differences between mutations that reduce HLA-A binding and mutations that do not.

To test the first hypothesis, we examined whether the observed dependence of the binding effect of single mutations on their fitness becomes more prominent if the frequency of HLA-A alleles are taken into account. To this end we defined a ‘mutation impact’, 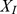, which calculates the unweighted binding impairment to all HLA molecules which bind to the wild-type, and a ‘population-weighted mutation impact’, 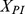, which calculates the same effect but weighted by the frequencies of particular HLA alleles (see Materials & Methods). A significant correlation between 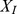 and fitness was observed (absolute criterion: 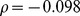, 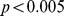; relative criterion: 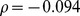, 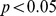), as well as between 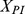 and fitness (absolute criterion: 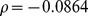, 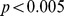; relative criterion: 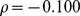, 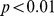), however the two correlation coefficients were not significantly different (absolute criterion: 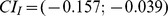, 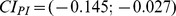; relative criterion: 
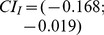
, 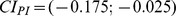). Furthermore, a partial correlation between fitness and 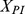 corrected for 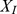, was found to be non-significant (absolute criterion: 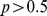; relative criterion: 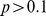). Hence, our analysis shows that the lower replicative fitness of mutations which impair binding to HLA-A molecules is unlikely to be caused by the population effect explained above.

**Figure 3:**
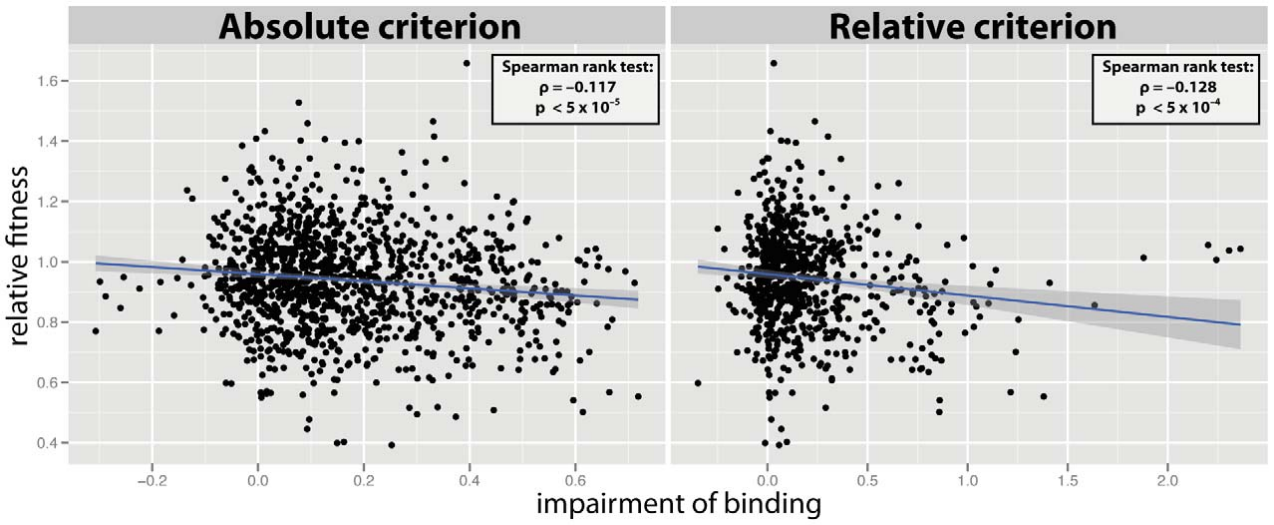
Cost of escape from rare HLA-A alleles. Correlation between fitness of single mutations and impairment of binding to rare (lower than 0.5%, see Materials & Methods) HLA-A molecules (absolute criterion: 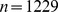, relative criterion: 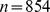). We observed a significant correlation for both epitope definitions (absolute criterion: 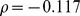, 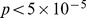; relative criterion: 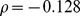, 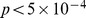). For the sake of illustration, the blue line shows the best fit of a linear regression and the 95% confidence interval.

To test the second hypothesis (intrinsic effect), we considered only escape mutations from rare HLA-A alleles (less frequent than 0.5%) because such rare alleles are likely to impose a negligible selective pressure on the virus population. Fig. 3 shows a correlation between fitness and impairment of binding due to single mutations in regions targeted by such infrequent HLA-A alleles, in analogy to Fig. 1. For both definitions of escape, we saw a significant correlation between fitness and impairment of binding affinity (absolute criterion: 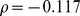, 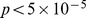; relative criterion: 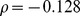, 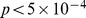). Our analysis thus suggests that the association between replicative cost and escape from HLA-A-binding in HIV-1 may be due to intrinsic differences between the mutational effects of escape versus non-escape mutations.

**Figure 4:**
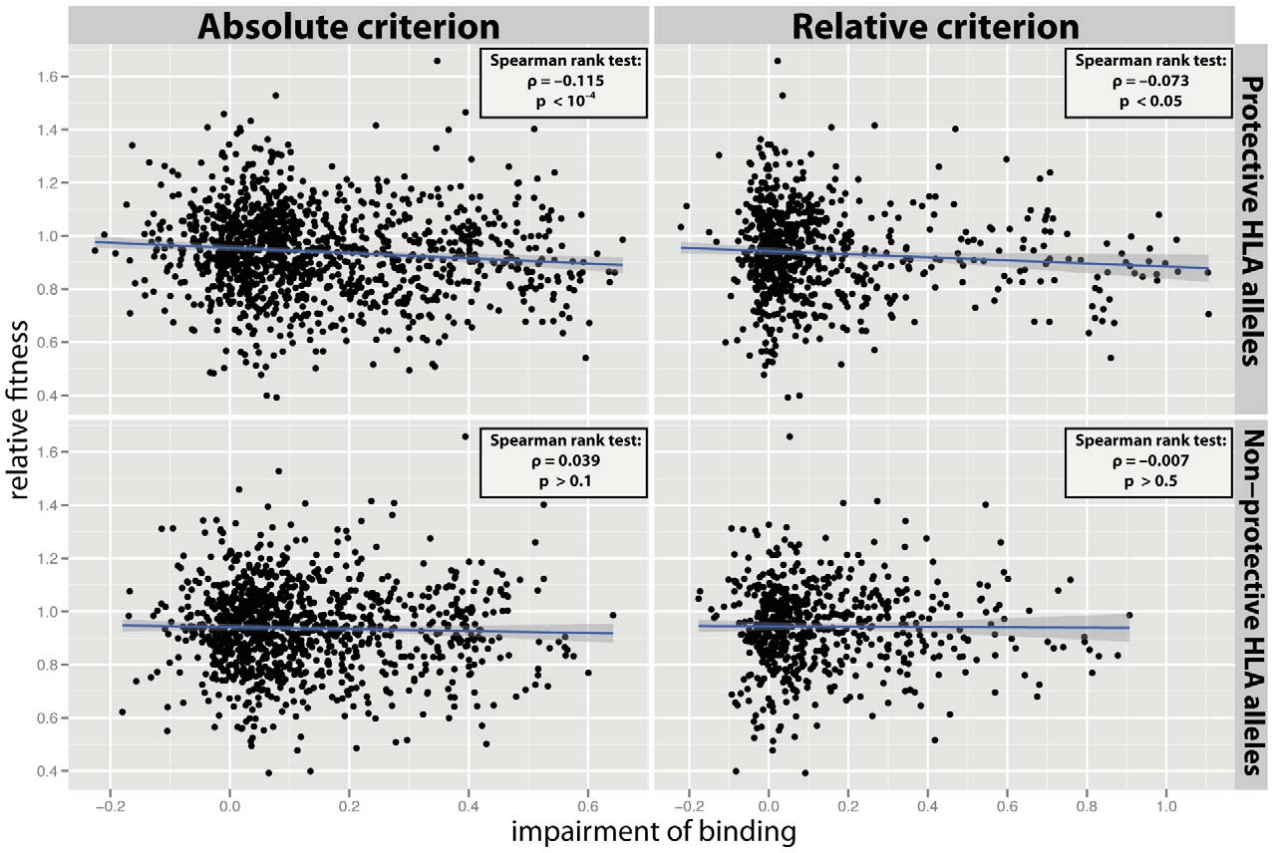
Cost of escape from the protective and the non-protective HLA alleles. (**Top row**) Effect of mutations on binding affinity to 10 most protective HLA molecules according to two alternative epitope definitions (absolute criterion: 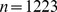, relative criterion: 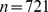). Protectiveness of alleles was characterized based on the relative hazard for AIDS progression (see Materials & Methods). A significant correlation between the fitness of single mutants and the impairment of binding to the most protective HLA molecules was observed (absolute criterion: 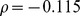, 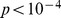; relative criterion: 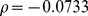, 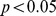). (**Bottom row**) Effect of mutations on binding affinity to 10 most non-protective HLA molecules according to two alternative epitope definitions (absolute criterion: 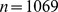, relative criterion: 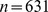). In this case, no significant correlation between the fitness of single mutants and the impairment of binding to the most non-protective HLA molecules was found (absolute criterion: 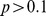; relative criterion: 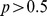). For the sake of illustration, the blue line shows the best fit of a linear regression and the 95% confidence interval.

Finally, we examined whether escape mutations from the presentation by protective HLA alleles are more costly than escape mutations from the presentation by non-protective HLA alleles. We defined protective and non-protective HLA alleles based on the associated relative hazard of progression to AIDS reported in an earlier study [46] (see also Materials & Methods). Fig. 4 shows a correlation between fitness and binding, in analogy to Fig. 1, for the 10 most protective and the 10 most non-protective HLA allele groups. A significant correlation is observed in the case of protective HLA alleles (absolute criterion: 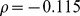, 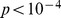; relative criterion: 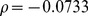, 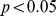) but not in the case of non-protective HLA alleles (absolute criterion: 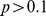; relative criterion: 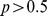). Furthermore, we observed that the correlations for protective HLA alleles became stronger when performed on the subset of protective HLA-A alleles (absolute criterion: 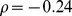, 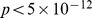; relative criterion: 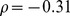, 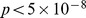), and non-significant when performed on the subset of protective HLA-B alleles (both criteria: 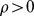, 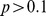). An analysis based on a dataset from a different study by Gao et al. [49] revealed qualitatively identical results. Our results thus suggest that escape from protective HLA alleles (particularly HLA-A alleles) may be more costly than escape from non-protective HLA alleles.

## Discussion

This study provides, to our knowledge, the first quantitative assessment of the fitness cost of escape mutations which disrupt presentation by HLA-A and HLA-B molecules. Our results indicate that the fitness cost of mutations increases with the resulting impairment of binding strength to HLA-A, suggesting that a higher tendency to escape HLA binding is more costly in terms of replicative fitness.

Our analysis suggests that the increased replicative cost of those mutations which tend to impair binding to HLA-A molecules is due to an intrinsic property of the virus, where HLA molecules preferentially bind peptides in which escape mutations are particularly costly to change, and this is not biased by the fact the analysis was done on clinically derived sequences (cf. Fig. 2). This intrinsic property of HLA binding is reflected as a significant difference between fitness cost and change in binding to rare HLA-A alleles, which are unlikely to exert any selective pressure at the population level (see Fig. 3). Furthermore, we found that mutations which help escape the protective HLA alleles (particularly HLA-A alleles) are associated with a higher fitness cost, unlike mutations which help escape the non-protective HLA alleles (see Fig. 4). This result also points to an intrinsic cost of escape as the protective HLA alleles used in our analysis are not only on average less frequent than the non-protective alleles, but also less frequent than all other alleles used in this study (protective: 0.56%; non-protective: 0.61%; all: 0.74%). We tested whether certain biochemical properties of the peptides, such as hydrophobicity [50], [51], amino acid similarity [52] or G
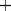
C content [53] could potentially underlie the observed intrinsic property of MHC binding. However, even though such properties may partially explain changes in binding to HLA molecules (e.g., change in hydrophobicity according to [54], [55]; see Fig. S1), none of them significantly correlates with changes in fitness (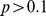). Therefore, what mechanism could explain that mutations which help to escape HLA-A binding are associated with a replicative cost, and whether intrinsic costs occur in other regions of the HIV-1 genome, remain open questions.

One of the most puzzling outcomes of this study is that no significant fitness costs have been observed for escape mutations from HLA-B molecules. This is surprising because HLA-B molecules have been shown to have the strongest impact on the outcome of HIV-1 infection: some HLA-B alleles have been associated with long-term non-progression to AIDS [16], [56], [57], and HLA-B restricted CTL responses have been shown to exert the strongest selective pressure on the virus [11], [58], [59]. In line with these observations, it has been previously shown that HLA-B alleles target more conserved genetic regions of HIV-1 than HLA-A alleles [60], [61]. However, the fact that we do not observe a significant cost of escape mutations from HLA-B can be potentially attributed to several, mostly methodological factors. First, a general feature of the currently available prediction software is that binding predictions are more accurate for HLA-A than for HLA-B, and this is also the case for netMHCpan used here [37]. Second, the differences in fitness costs between HLA-A and HLA-B could be due to an unknown property of the HIV-1 genomic region considered (PR and RT). Third, because the mutations analyzed here are extracted from clinically derived sequences, it is conceivable that the lack of a significant difference between fitness of escape and non-escape mutations for HLA-B is due to the differences between the distributions of HLA-A and HLA-B alleles in the North American population. This topic, however, warrants further investigation. For the reasons outlined above, our results should not be used to suggest that escape from HLA-A presentation is more costly than escape from HLA-B presentation. Rather, our study emphasizes an important role of HLA-A alleles (particularly the protective ones) in the evolution of HIV-1, and RNA viruses in general, as suggested previously [62]–[65].

One of the limitations of this study is that we have consistently ignored all mutations outside the regions predicted to be restricted by any HLA alleles used in the analysis (for the list see Table S1). Interestingly, when we compared the fitness of mutations restricted by HLA-A molecules to those not predicted to be restricted by any of the HLA-A molecules used in this study, we found that mutations in the restricted regions are on average less costly than mutations in the non-restricted regions (absolute criterion: 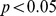, relative criterion: 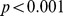), and a similar effect was found for HLA-B molecules (absolute criterion: 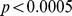, relative criterion: 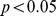). The difference in fitness cost of the restricted versus non-restricted regions may seem to contradict our earlier conclusion that HLA-A molecules target regions which are more costly for the virus to change. However, as the extent of those regions strongly depends on the number of HLA molecules considered in the analysis, as well as the binding threshold considered, this result needs to be interpreted with caution. For example, in the case of the relative criterion, we find that HLA-A molecules span 60% and HLA-B span 67% of the considered HIV-1 PR and RT, and for the absolute criterion these numbers are even larger (85% and 98%, respectively). Even though we used the largest subset of HLA molecules for which the employed binding predictor works and for which frequency data in the US population were obtained, these numbers constitute only a fraction of the actual numbers of HLA alleles in the human population (98 out of known 486 HLA-A alleles and 184 out of known 817 HLA-B alleles) [16]. It therefore seems likely that the non-restricted regions here (i.e., those regions in which no peptide binds to any of the HLA molecules included in the study) do bind to other HLA molecules not included in this analysis, however this hypothesis remains to be tested.

Another caveat of this study is that the viral fitness has been assessed in an *in vitro* assay. This may be partially responsible for the seemingly counter-intuitive fact that many single mutations increase viral replicative capacity, as seen e.g. in Fig. 1. Nevertheless, a recent study has shown that *in vitro* fitness measures in PR and RT are indicative of *in vivo* HIV-1 virus load, suggesting that our fitness measures are justified as a proxy for the virus fitness *in vivo* [66].

It should also be noted that the correlations we observe throughout this study are generally weak, suggesting that escape from HLA-A presentation explains around 10% of the variance in viral fitness. This may seem surprising as HLA molecules have been shown as a potent factor in the evolution of HIV-1 [16], [17], [67], [68]. However, there are several reasons to expect such a result. First, HLAs are probably not the sole driver of the evolution of the virus. Many of the mutations predicted to be non-escape mutations could actually be escape mutations from other elements of the antigen-processing and presentation pathway (e.g., TAP, proteasome, MHC class II), or from the recognition by the T cell receptors [3]. Second, many epitopes are known to vary in their immunogenicity, and some will be more likely to elicit CTL responses than others [69]. Third, the signature of evolution in our dataset will be strongly influenced by the population structure from which the data was obtained, e.g., the distribution of HLA allele frequencies. Fourth, the Pol gene analyzed here plays an important role in the evolution of drug resistance, leading to a potential interaction between immune- and treatment-mediated selection (see below). Finally, the dataset used cannot provide any insight into the distribution of the impairment of binding of mutations with extremely high replicative fitness costs, because such mutations will only be found at very low frequencies in the patient population and may therefore be completely absent in the dataset underlying our analysis. Overall, it is plausible that even if CTL-mediated pressure is as strong as suggested previously [26], [70], [71], many other factors (in addition to escape from HLA class I) have influenced the evolution of HIV-1, resulting in a weak correlation between replicative fitness and change in binding to HLA molecules.

Can the implications of this analysis be extrapolated to the entire HIV-1 genome? Previous studies showed that the Pol gene, even though not as immunodominant as the Gag gene, can still play an important role in the interactions with CTL-mediated immunity [11], [72]–[74], and many optimal CTL epitopes have been identified in the genetic region examined here [48]. Therefore, it is conceivable that the effects observed here may be even stronger in the most immunodominant HIV-1 proteins, like p24 Gag. Related to this point is the fact that the gene analyzed here gives rise to many known drug resistance mutations. Even though our results were qualitatively identical when all such known mutations were excluded [75], we observed an interesting interplay between *in vitro* fitness and binding impairment for the subset of known drug resistance mutations. In particular, we found a significant negative correlation between fitness and binding impairment of those mutations to HLA-A molecules for one of the criteria (absolute criterion: 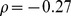, 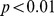; relative criterion: 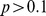), and a trend of a positive correlation between fitness and binding impairment of those mutations to HLA-B molecules (absolute criterion: 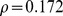, 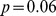; relative criterion: 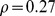, 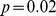). This could point to an interesting interplay between the evolution of resistance and the evolution of escape in HIV-1, as emphasized previously [76]. However, the exact character of this relation remains unclear.

Assessing the fitness cost of immune escape mutations presents a crucial step towards a quantitative understanding of the dynamics of infectious diseases and their interactions with the immune system. To our knowledge, this study represents the first attempt to quantify the cost of mutations on a large scale and to compare it with the cost of mutations which do not affect the interaction with the immune system. The fact that a relation between fitness cost of mutations and their propensity to confer escape is not caused by a population effect in the analyzed data suggests that MHC class I molecules might have evolved to bind the genetic regions of RNA viruses which are costly to change.

## Supporting Information

Figure S1Hydrophobicity vs. impairment of binding to HLA alleles. Change in hydrophobicity (hydrophobicity of the mutant amino acid minus hydrophobicity of the wild-type amino acid) was correlated with the maximal impairment of binding to both HLA-A and HLA-B alleles (see main text). A significant negative correlation was found for all three measures of hydrophobicity and for both binding criteria used (absolute criterion: 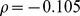, 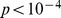 [measure 1], 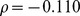, 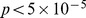 [measure 2], 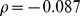, 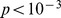 [measure 3]; relative criterion: 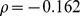, 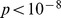 [measure 1], 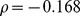, 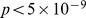 [measure 2], 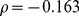, 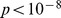 [measure 3]). The new consensus hydrophobicity scale was used as measure 1 (Tossi et al., 2002), the pH 7.4 hydrophobicity scale was used as measure 2, and the pH 2.1 hydrophobicity scale was used as measure 3 (Meek, 1980). Even though the results shown here are obtained for both HLA-A and HLA-B, the results for only HLA-A and only HLA-B were qualitatively identical. For the sake of visibility, the blue line shows the best fit of a linear regression with 95% confidence interval.(TIF)

Table S1 The table lists all HLA alleles (A and B) used in the analysis. The first row lists the HLA alleles in the four-digit precision. The second row gives their expected frequency in the US population (see also Materials & Methods).
(XLS)
