# Supplementary figures and images for: Estimating the Fitness Cost of Escape from HLA Presentation in HIV-1 Protease and Reverse Transcriptase
Source: PLoS Comput Biol. 2012 May 24;8(5):e1002525. doi: 10.1371/journal.pcbi.1002525 (PMC3359966; doi:10.1371/journal.pcbi.1002525)

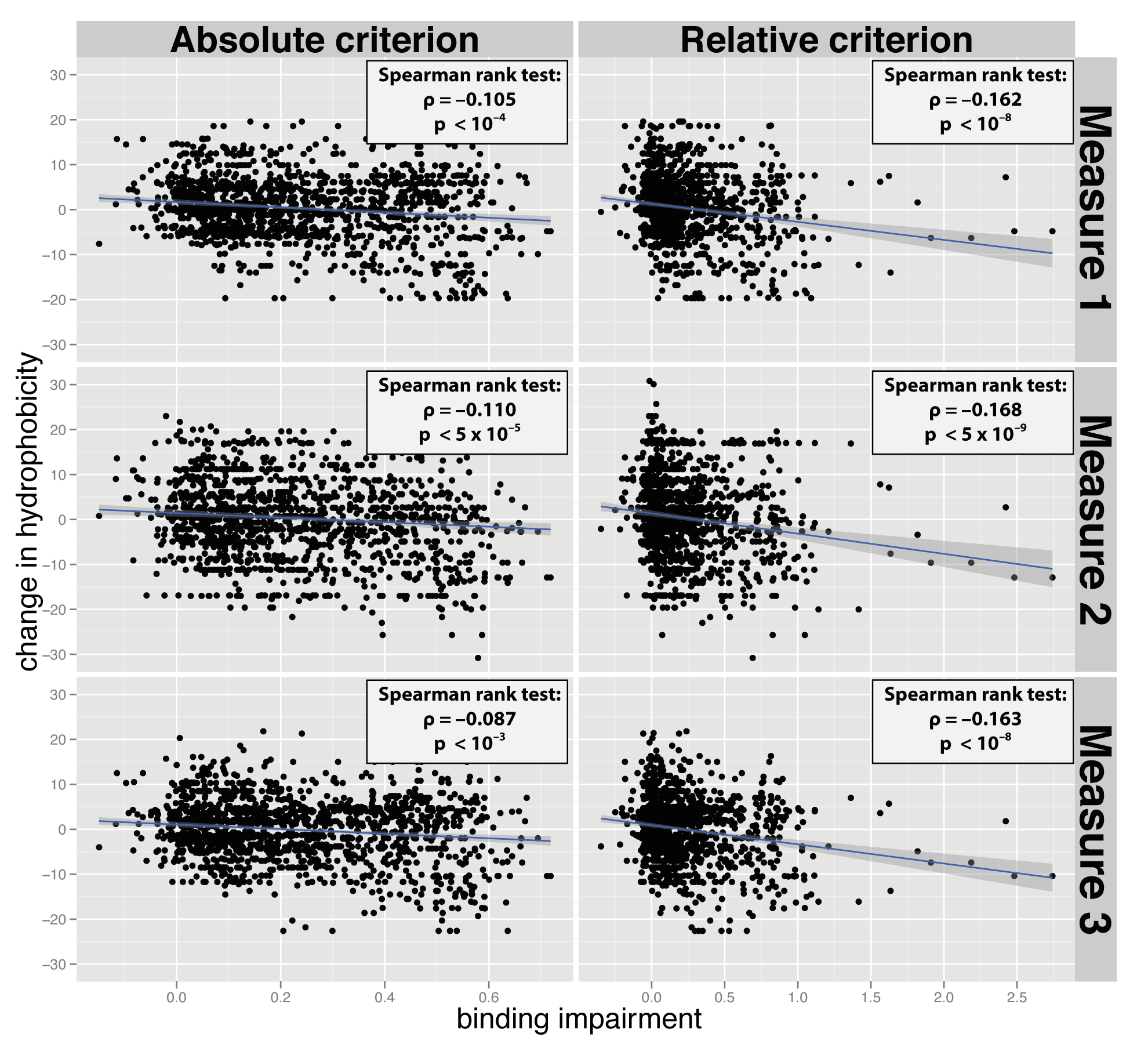

Supplement: Figure S1 — Hydrophobicity vs. impairment of binding to HLA alleles. Change in hydrophobicity (hydrophobicity of the mutant amino acid minus hydrophobicity of the wild-type amino acid) was correlated with the maximal impairment of binding to both HLA-A and HLA-B alleles (see main text). A significant negative correlation was found for all three measures of hydrophobicity and for both binding criteria used (absolute criterion: , [measure 1], , [measure 2], , [measure 3]; relative criterion: , [measure 1], , [measure 2], , [measure 3]). The new consensus hydrophobicity scale was used as measure 1 (Tossi et al., 2002), the pH 7.4 hydrophobicity scale was used as measure 2, and the pH 2.1 hydrophobicity scale was used as measure 3 (Meek, 1980). Even though the results shown here are obtained for both HLA-A and HLA-B, the results for only HLA-A and only HLA-B were qualitatively identical. For the sake of visibility, the blue line shows the best fit of a linear regression with 95% confidence interval. (TIF) [file pcbi.1002525.s001.tif]
